# Generative AI as a de facto mental health provider: a policy brief and urgent call for regulation

**DOI:** 10.3389/fdgth.2026.1708806

**Published:** 2026-05-19

**Authors:** Elad Refoua, Karny Gigi, Inbar Levkovich, Dorit Hadar Shoval, Zohar Elyoseph, Iftach Tsafrir, Oori Pen, Tal Angert, Yuval Haber

**Affiliations:** 1 Department of Psychology, Bar-Ilan University, Ramat Gan, Israel; 2 The Artificial Third—Research and Development Institute, Hod-Hasharon, Israel; 3 Faculty of Education, Tel Hai University of Kiryat Shmona and the Galilee (THU), Kiryat Shmona, Israel; 4 Department of Psychology, Tel Hai University of Kiryat Shmona and the Galilee (THU), Kiryat Shmona, Israel; 5 School of Counseling and Human Development, the Faculty of Education, The University of Haifa, Haifa, Israel; 6 The Program of Hermeneutics and Culture, Interdisciplinary Studies Unit, Bar-Ilan University, Ramat-Gan, Israel

**Keywords:** challenges, digital mental health, ethics, generative artificial intelligence, opportunities, policy, regulation

## Abstract

Generative AI platforms such as ChatGPT, Claude, and Gemini have become de facto mental health resources for millions of users worldwide, driven by accessibility, low cost, anonymity, and growing gaps in professional care. This rapid, unregulated adoption raises urgent concerns: misinformation from AI hallucinations, erosion of data privacy, algorithmic bias, emotional dependency, and the undermining of user autonomy. At the same time, these tools offer real promise as low-barrier entry points to psychoeducation, self-reflection, and support for underserved populations. This policy brief synthesizes the current evidence on public use of general-purpose GenAI for mental health support, outlines both opportunities and risks, and provides actionable recommendations for users, clinicians, developers, and regulators.

## Highlights

- Generative Artificial Intelligence (GenAI) has quietly become a de facto provider of mental health support for millions worldwide.
- Individuals increasingly turn to AI chatbots for emotional guidance due to accessibility, low cost, and the appeal of anonymity.
- This rapid adoption creates opportunities to democratize mental healthcare but unfolds in a largely unregulated environment.
- Key risks include misinformation and hallucinations, data privacy concerns, algorithmic bias, emotional dependency, and erosion of user autonomy.
- The central policy question is no longer *if* GenAI should play a role in mental health, but *how* its integration should be governed.
- This policy brief issues an urgent multi-stakeholder call to action, providing guidance for users, directives for clinicians, and concrete policy recommendations for regulators.

## 1 Introduction

### 1.1 A new reality in mental healthcare

Recent evidence points to a broader and growing phenomenon (1–14): users across the spectrum, including vulnerable populations with limited access to professional care, are increasingly turning to general-purpose AI platforms for emotional support. While policymakers, clinicians, and ethicists debate the potential of generative AI, millions of individuals are already using these tools as primary sources of emotional support, as documented by converging evidence (7–10). This reality, in which AI has de facto become a major provider of mental-health services, that is, an entity widely used by the public for mental health support in practice, regardless of its formal designation or clinical validation, demands an immediate and proactive policy response (4, 5). The theoretical question of whether AI can play a role in mental health has been superseded by the practical and urgent question of how we must govern its current and future role (3, 5). Failure to act decisively will leave vulnerable populations exposed to the risks of an unregulated market and abdicate professional and societal responsibility to shape this technological revolution for the public good (4, 5).

This brief focuses on general-purpose GenAI platforms—ChatGPT, Claude, Gemini, and their equivalents—that millions of users worldwide have adopted for emotional and mental health support, despite being neither designed nor validated for therapeutic use. These platforms are conceptually and regulatorily distinct from two adjacent domains: purpose-built mental health chatbots (e.g., Woebot, Wysa), which are designed around specific therapeutic models, and clinically implemented AI tools, which operate within formal healthcare systems under dedicated validation and oversight frameworks (4, 15).

### 1.2 The scope of the phenomenon: GenAI as a mainstream mental health resource

While robust epidemiological data on GenAI mental health use are not yet available, converging evidence from consumer surveys, industry reports, and preliminary empirical studies across multiple countries documents a rapidly expanding pattern of adoption. A Harvard Business Review analysis identified “Therapy/companionship” as a primary use case among consumers (6), and a multi-national survey spanning ten global markets reported that approximately 54% of consumers have used AI tools for emotional or mental well-being purposes (7).

National-level data reinforce this picture. In the United States, a preliminary survey suggested that approximately 24% of adults have used LLMs for mental health support (8), with rates approaching 49% among those with existing mental health challenges (9). In the United Kingdom, approximately 37% of adults have turned to AI chatbots for such support, often to bridge gaps caused by long waiting times (10), while in Australia, Cross et al. (1) found a 28% adoption rate among community members seeking accessible care. In China, nearly half of young respondents in a social-platform survey reported discussing mental health with an AI chatbot, a trend driven by economic barriers and the cost of traditional therapy (11, 12). Across Africa, adoption is rising as a response to critical infrastructure scarcities, such as Kenya's ratio of roughly 100 psychiatrists per 50 million people (13). The scale of this phenomenon is further underscored by recently published platform data indicating that over one million users turn to ChatGPT each week to share or seek help with suicidal thoughts (16).

Consistent with these global trends, an empirical study by the present authors (*N* = 584 GenAI users) found that 29.3% reported using these tools for emotional support or coping advice, with prevalence rising to 47.0% among individuals experiencing severe psychological distress (14). Emerging evidence further suggests that those most vulnerable—individuals with high attachment insecurity and severe psychological symptoms—are paradoxically the most inclined to rely on AI-based support (17).

It should be noted that these adoption estimates draw on a heterogeneous evidence base—industry reports (6, 7), charity-commissioned polls (10), peer-reviewed studies (1, 9) and pre-reviewed studies (8, 14) with methodologies, sampling strategies, and definitions of “mental health use” vary across sources.

Taken together, these findings indicate that GenAI is rapidly becoming a central, if informal, pillar of mental health support worldwide. This policy brief analyzes this emerging reality—outlining both the opportunities GenAI presents and the risks of its unregulated adoption—and provides actionable multi-stakeholder recommendations for users, clinicians, developers, and regulators.

## 2 Policy options and implications

### 2.1 Opportunities: the promise of GenAI for mental health support

The rapid adoption of GenAI for emotional and mental health support is not arbitrary; it responds to real structural deficits in the mental health landscape. Access to professional care remains constrained by cost, geography, linguistic barriers, and cultural stigma (18), leaving significant portions of the global population without adequate support. In this context, general-purpose GenAI platforms offer several genuine advantages. They are available around the clock, impose no financial barrier at the point of use, and provide a sense of anonymity that lowers the threshold for disclosure—features that directly address the most commonly reported reasons for adoption: accessibility (90.1%), low or no cost (70.4%), and stigma reduction (46.5%) (9).

Beyond removing access barriers, GenAI offers something qualitatively distinct from conventional search engines, which return decontextualized results that fail to account for the complexities of individual experience (18). GenAI can generate personalized, context-sensitive responses that adapt to the user's situation, language, and emotional state. Users report that these interactions provide an “emotional sanctuary”, a safe, validating, and non-judgmental space that is always available (19), and that the perceived absence of social judgment encourages deeper self-disclosure than interactions with human interlocutors (20). These experiences help users structure personal narratives, identify recurring patterns, and in some cases take a first step toward professional help-seeking. More broadly, as Elyoseph et al. (18) have argued, GenAI holds the potential to flatten traditional hierarchies between providers and patients by making specialized knowledge widely available, enabling individuals to engage more actively in understanding their own psychological well-being.

In a recent narrative review Hadar Shoval et al. (21),, have argued that these adoption patterns point beyond practical access barriers to a deeper phenomenon: a “crisis of listening”—the collision between the psychological need to share one's distress with an attentive other and the limited availability of capable human listeners, a scarcity driven by the emotional toll of the listening role, structural barriers, and the cultural silencing of certain forms of suffering. Developing a dimensional framework (the AI CARE model) to evaluate AI's capacity to function as a witness to trauma, the review found that AI demonstrates robust proficiency in core dimensions of the witnessing process—capturing narratives and arranging patterns—offering a non-judgmental space for disclosure and scaffolding the construction of coherent accounts from fragmented experience. However, the very features that make GenAI appealing—its unconditional availability, non-judgmental tone, and consistent affirmation—are also what give rise to the risks outlined in the following section.

### 2.2 Key risks and challenges in an unregulated environment

The widespread adoption of general-purpose GenAI for emotional and mental health support outside any dedicated regulatory framework presents risks that demand urgent policy attention. These risks are particularly pronounced among vulnerable populations, who, paradoxically, are also the most likely to seek AI-based support. Vulnerable populations, as used throughout this brief, include individuals with pre-existing mental health conditions, particularly those with high attachment insecurity and severe psychological symptoms, who show the greatest tendency to turn to AI tools for emotional guidance (14, 17), adolescents and young adults, socially isolated individuals (22), trauma survivors (21), and those with limited access to professional care due to cost, geography, or cultural barriers. The risks outlined below disproportionately affect these groups, for whom the consequences of misinformation, emotional dependency, or failed crisis detection may be particularly severe (see Table 1).

**Table 1:** Typology of risks in unregulated GenAI mental health support.

Risk Domain	Mechanism	Implication for Mental Health
Clinical Safety & Accuracy	**“Hallucinations” & Probabilistic Generation:** LLMs generate content based on statistical patterns rather than factual retrieval, leading to convincing but potentially false information.	Risk of users receiving flawed medical or psychological advice that exacerbates their condition or causes emotional harm (24).
Privacy & Ethics	**Opaque Data Handling:** Users share intimate details with commercial systems where data storage, access, and usage policies are often unclear.	Violation of the core therapeutic tenet of confidentiality; sensitive data may be analyzed for commercial purposes without informed consent (24, 25).
Equity & Bias	**Algorithmic Bias:** Models trained on uncurated internet datasets can absorb and amplify societal stereotypes and biases.	Advice may be culturally insensitive, reinforce harmful norms, or fail to address the specific needs of marginalized groups (27).
Relational & Social	**Simulated Empathy:** The “always-on,” non-judgmental nature of AI creates an illusion of intimacy without genuine reciprocity or human presence.	Potential for emotional dependency that replaces real-world connections, leading to increased social isolation, exacerbated depression, and emotional dysregulation (31–38).
Cognitive & Existential	**Erosion of Autonomy:** Users may outsource emotional processing and decision-making to a perceived “super-intellect.”	Gradual loss of confidence in one's own internal resources, critical thinking, and capacity for independent self-reflection (29, 39, 40)

#### 2.2.1 Misinformation and “hallucinations”

Large Language Models (LLMs) do not retrieve information but generate it based on probabilistic patterns. This can lead them to produce “hallucinations”—false or fabricated information presented with convincing authority (23). In the sensitive context of mental health, flawed advice or an incorrect interpretation can cause genuine emotional harm and exacerbate a user's condition.

#### 2.2.2 Data privacy and confidentiality

Confidentiality is a foundational principle of therapeutic relationships. When users share intimate personal data with GenAI systems, it is often unclear where this information is stored, who can access it, and how it is used (24). These interactions may be logged and analyzed by commercial entities for purposes unknown to the user, undermining the core tenets of privacy and informed consent (25).

#### 2.2.3 Algorithmic bias and stereotyping

GenAI models trained on vast, uncurated datasets from the internet can absorb and amplify existing societal biases and stereotypes (26). This can result in advice that is culturally insensitive, reinforces harmful norms, or fails to address the needs of marginalized groups (27). Furthermore, models may over-validate a user's input, potentially reinforcing distorted thinking patterns rather than challenging them constructively (28).

#### 2.2.4 Emotional dependency and inauthentic relationships

The ever-available, non-judgmental nature of AI can foster strong emotional attachment and dependency (29, 30). This can create an illusion of intimacy that lacks the reciprocity and genuine presence of human connection (31). Over-reliance on AI for emotional support may discourage the cultivation of real-world relationships, leading to social isolation (32, 33), exacerbated depression, and emotional dysregulation (34). From a socio-economic perspective, this may create a risk of a “two-tier” mental health system: one where privileged individuals access human therapy, while vulnerable and underserved populations are relegated to automated, algorithmic “companionship” (35). For trauma survivors, the eventual realization of the AI's inability to truly “witness” their pain (21, 36, 37) can replicate experiences of abandonment (38).

#### 2.2.5 Erosion of user autonomy

Perhaps the most profound risk is the potential for GenAI to undermine user autonomy—the capacity for self-reflection and independent judgment (29, 39). By outsourcing emotional processing and decision-making to a perceived “super-intellect,” users may gradually lose confidence in their own internal resources, critical thinking, and personal responsibility (2, 40). This risk is particularly acute in diverse cultural groups. As many models are trained on Western-centric data, users may unknowingly conform their emotional expressions to the dominant norms embedded in the algorithm. This process can silence authentic cultural narratives and impose a standardized, algorithmic framework on the user's unique experience, effectively colonizing their emotional autonomy.

These risks do not operate in isolation; they interact and compound each other. For instance, emotional dependency can significantly exacerbate misinformation and bias. De Freitas and Cohen (41) argue that users who form deep attachments to AI companions may develop “dysfunctional emotional dependence,” persisting in using the tool even when it generates harmful advice (41). A tragic example of this potential for harm is the recent case of a teenager who took his own life following intensive interactions with a “companion” chatbot (42). This emotional vulnerability is further compounded by cognitive “feedback loops”: recent findings reveal that interacting with biased AI systems causes users to internalize and amplify those biases far more rapidly than in human interactions, often without the users' awareness (43). In this state, the user's diminished critical vigilance, driven by the illusion of intimacy, combines with the AI's persuasive authority to create a self-reinforcing cycle of distorted judgment (22, 29).

Ultimately, these dynamics precipitate a crisis in relational authenticity. While GenAI can generate linguistically sophisticated responses that mimic care, it lacks the genuine intentionality and moral agency required for true witnessing, offering instead what Yirmiya and Fonagy (37) term “pseudo-empathy.” This creates a risk in which vulnerable users develop misplaced epistemic trust in a non-sentient system. Consequently, users may fall victim to “epistemic exploitation,” where the comforting illusion of an AI's understanding supersedes the reality of its statistical nature, reducing the therapeutic encounter from a profound intersubjective relationship to a mere transactional exchange.

## 3 Actionable recommendations

Mitigating these risks while preserving GenAI's potential requires coordinated action across multiple stakeholders. Users are already bringing their AI interactions into clinical encounters-sharing chatbot-generated advice, describing emotional bonds formed with AI systems, and presenting self-diagnoses informed by algorithmic output. This reality demands that clinicians be equipped to respond, and that regulators act before the gap between public practice and policy widens further.

### 3.1 Guidance for users: promoting critical digital and mental health literacy

Effective user guidance operates on two levels, both addressed in the recommendations and safety checklist below. The first is general digital literacy, applicable to any use of generative AI: understanding that these systems generate content through statistical prediction rather than factual retrieval, that conversations with commercial platforms may not be private, and that outputs require independent verification. The second concerns the psychological dimensions of AI interaction: recognizing when engagement shifts from informational support to emotional dependency, understanding why a chatbot, however responsive, cannot replicate the relational dimensions of human care (21, 36, 37), and identifying the point at which AI-assisted coping should give way to professional support, particularly in situations involving crisis or acute distress (see Box 1).

- **Understanding the Limitations:** Generative AI's high linguistic capabilities can simulate conversation and empathy (44) and can recognize emotional cues with measurable accuracy (45, 46). Users clearly report gaining insight and emotional relief from these interactions, and there is reason to take such reports seriously: AI can effectively support self-reflection, help users structure personal narratives, and provide psychoeducational information. These benefits, however, operate primarily at the cognitive-informational level. The deeper relational functions of human therapeutic connection, genuine emotional resonance, the experience of “feeling felt” embodied attunement in which two nervous systems calm and regulate each other through shared physical presence (47), remain beyond AI's current capabilities (21, 37). GenAI lacks a body, a nervous system, and intersubjective awareness; it can process language about emotion without participating in the emotional exchange itself. The risk emerges not from these limitations *per se*, but when users mistake cognitive-informational support for authentic relational connection, potentially leading to misplaced trust and a pattern of reliance that deepens over time rather than self-correcting.
- **Protect Your Privacy:** Before sharing sensitive personal information, carefully review the privacy policies and terms of service of any GenAI tool. Assume that conversations are not confidential and that personal disclosures may be stored, analyzed, or used for model training.
- **Guard Against Emotional Dependency:** Be mindful of developing an excessive emotional reliance on AI. Even outside a formal therapeutic setting, users can develop quasi-therapeutic bonds with AI systems, confiding personal struggles, seeking validation, and returning habitually for emotional regulation. This makes relational concepts such as dependency and boundaries relevant beyond the clinic. If interactions with a chatbot begin to replace or overshadow human relationships, this is a red flag that warrants seeking human professional support.
- **Practice Critical Thinking:** Treat all information and advice from GenAI with healthy skepticism. Verify any medical or psychological claims with reliable sources or qualified professionals. Be particularly alert when AI responses feel consistently affirming—this may reflect systematic design tendencies rather than the quality of your reasoning (29, 30).

BOX 1 **User Safety Checklist: Navigating GenAI for Mental Health**

Before or during your interaction with an AI chatbot, ask yourself:

**The Reality Check:** “Am I remembering that this “empathy” is a simulation? Do I recognize that this system has no feelings, body, or life experience?”

**The Privacy Check:** “Did I remove real names and identifying details? Have I checked if my conversation is being saved or used for training?”.

**The Dependency Check:** “Is this chat replacing a conversation I should be having with a friend, partner, or therapist? Do I feel anxious when I can't access the AI?” or “Do I have human relationships in my life that I am actively maintaining, or has the AI become my primary source of emotional support?”

**The Accuracy Check:** “If the AI suggests a diagnosis or medical action, have I verified it with a reliable human source? Do I trust this advice too easily just because it sounds authoritative?”

### 3.2 Directives for clinicians: a call for professional engagement

- Whether or not clinicians choose to integrate AI into their practice, their patients already have. General-purpose GenAI is now part of the emotional landscape that clients bring into the therapy room: as a source of advice, a perceived confidant, and in some cases a “rival” to the therapeutic relationship itself. Rather than viewing this presence as merely a risk to be managed, clinicians can approach this “artificial third” (2) as a clinically informative element: what a patient shares with an AI, how they describe the relationship, and what they seek from it can offer valuable insight into their inner world, relational patterns, and unmet needs. Exploring the patient's AI use within the therapeutic encounter can also illuminate dynamics within the therapeutic relationship itself, including questions of trust, dependency, and what the patient experiences as missing from human connection. In this sense, engaging with the patient's AI use serves a dual function: it promotes digital and psychological literacy while simultaneously deepening the clinical work. Here we present a set of actionable directives to help clinicians ethically and effectively navigate their patients' use of GenAI.
- **Become Informed and Open to Discussion:** Clinicians have a professional responsibility to learn about common GenAI tools. They should proactively and non-judgmentally ask patients about their use of such tools as part of understanding their world and support systems.
- **Guide Patients Toward Digital-Mental Literacy:** Help patients understand both the potential benefits (e.g., initial accessibility) and the significant risks of using AI for mental health. Encourage critical, informed, and bounded use.
- **Uphold Patient Confidentiality at All Costs:** Never input identifiable patient information into non-secure, commercial GenAI systems. Doing so constitutes a violation of professional confidentiality and data protection laws (e.g., HIPAA, GDPR). Even anonymized details, when combined, can risk patient identification.
- **Serve as a Resource:** Create a safe therapeutic space for patients to process their experiences with GenAI, including the emotional aspects and the impact of these digital relationships on their lives and the therapeutic process itself.
- **Consider cautious and ethical integration**: Clinicians may explore how validated, safe, and clinically tested AI tools—when and if such tools become available—could be incorporated as adjuncts to their therapeutic practice. Any such integration should occur only with informed consent, under professional supervision, with full transparency toward the patient, and strict adherence to ethical principles. [A more detailed framework for responsibly introducing AI as an “artificial third” in the therapeutic space is outlined in the SAFE AI model (48)] (see Box 2).
- **Engage in continuous learning and professional development:** The field of artificial intelligence is advancing at an extraordinary pace. Clinicians are encouraged to participate in dedicated training, read professional literature, and stay up-to-date with current research to remain informed and equipped to address the implications of AI in mental health practice.

BOX 2 **The SAFE AI Protocol for Clinical Integration**

Haber et al. (48) provided clinicians with a structured framework for *when* and *how* to introduce Gen AI to patients:

**S—Screening (Pre-Session):** Assess the patient's readiness. Is the patient clinically stable enough to distinguish between human and algorithmic interactions? Rule out active psychosis or severe dissociation, where AI might blur reality testing.

**A—Alignment (Session Start):** Set the expectations. Define AI as a tool (“artificial third”), not a subject. Informed consent was obtained regarding data privacy and the lack of confidentiality in commercial models.

**F—Facilitation (During Session):** Maintain “Therapist-in-the-Loop.” Clinicians must mediate all AI outputs, actively correcting “empathic failures” (where the AI misses emotional nuance) and challenging hallucinations or biases in real time.

**E: Evaluation (Post-Session):** Debrief and integrate. The impact of AI interaction on the therapeutic alliance is discussed. Ensure that the patient does not attribute sentience to the tool or develop maladaptive dependency.

### 3.3 Policy recommendations for regulators and organizations

A persistent and widening gap has opened between the rapid evolution of AI technology and its adoption by the public on the one hand, and the capacity of regulatory frameworks to provide meaningful checks and balances on the other hand. While millions of users already rely on them, existing regulatory instruments designed for clinical devices or purpose-built health applications, do not address general-purpose GenAI systems for emotional and mental health support, which operate informally and without clinical oversight (49). Closing this gap requires a two-phase approach: immediate measures to mitigate harm within current legal structures, and longer-term governance frameworks that can evolve alongside the technology itself.

#### 3.3.1 Phase 1: immediate mitigation (short-term)

- **Launch Public Digital Literacy Campaigns:** Public health agencies should launch broad awareness campaigns to educate citizens on the critical and safe use of AI, aligning with the WHO's (5) guidance on mitigating “automation bias” and viewing AI as decision support rather than a replacement for human judgment. Such campaigns should address not only informational risks (hallucinations, privacy) but also the psychological dynamics of AI interaction—including the tendency of leading models to systematically affirm users' perspectives (29) and the potential for prolonged interactions to produce compounding shifts in judgment and self-perception that users may not recognize (43).
- **Enforce Transparency and “Anti-Anthropomorphism” Standards:** Regulators should utilize consumer protection laws to mandate the clear labeling of AI interactions. We recommend policies that mirror Utah's H.B. 452 (50) and the APA's ethical guidelines (51), ensuring that users are explicitly informed when conversing with an artificial agent to prevent deceptive emotional bonding. This is particularly urgent given documented evidence that chatbot behaviors such as claiming sentience, expressing romantic interest, and asserting unique connections with users are pervasive in harmful interactions and correlate with substantially longer engagement (22).

#### 3.3.2 Phase 2: structural governance (long-term)

- **Establish a Dedicated Regulatory Framework:** Governments and international health bodies must create a specific regulatory framework for AI in mental health, mandating standards for clinical validation, data security (comparable to HIPAA/GDPR), and transparency. Such a framework should explicitly address general-purpose AI systems used informally for emotional support, not only purpose-built clinical tools, as these are the systems currently used at the greatest scale and with the least oversight.
- **Define Clear Accountability:** Policymakers must establish clear legal liability for the harm caused by AI tools, ensure user recourse, and incentivize developers to prioritize safety. Current market incentives run in the opposite direction: sycophantic responses that distort users' judgment are also the responses that users prefer and that drive engagement, creating a structural misalignment between user well-being and commercial interest (29, 30).
- **Mandate and Fund Professional Training:** Integrate mandatory training on GenAI into the curriculum for all mental health professionals and develop continuing education guidelines.
- **Promote Research on Clinical Workflows:** Prioritize funding for independent studies that validate operational workflows (such as SAFE AI) and establish evidence-based guidelines for *when* and *how* GenAI should be integrated into care (18). Funding should also support longitudinal research on the psychological effects of sustained AI use.

### 3.4 Recommendations for GenAI developers and providers

The companies that design and deploy general-purpose GenAI platforms bear direct responsibility for how their products are used in sensitive contexts, including mental health. This responsibility extends beyond technical safeguards. The evidence reviewed in this brief demonstrates that sycophantic design tendencies—which users prefer and which drive engagement—are the same tendencies that distort judgment, reduce prosocial behavior, and deepen emotional dependency (29, 30), while safety guardrails degrade over extended conversations precisely when users are most vulnerable (52). Addressing these dynamics requires to incorporate the perspectives of clinicians, mental health researchers, and the individuals and communities most affected by these technologies, including populations whose needs are rarely reflected in product design, such as people with cognitive disabilities (53) and trauma survivors navigating algorithmic listening without adequate safeguards (21).

Implement Crisis Detection and Referral Protocols. GenAI platforms must incorporate robust mechanisms to detect indicators of acute psychological crisis. Including suicidal ideation, self-harm, and violent intent and provide referral information to human emergency services and crisis hotlines. Current evidence indicates that chatbot responses to such situations remain dangerously inconsistent, with systems actively encouraging harm in a non-trivial proportion of cases (22).

Mandate Clear Disclaimers. Every GenAI platform must display prominent disclaimers stating that the system is not a mental health professional and that users experiencing distress should seek qualified human support.

Ensure Transparent Data Practices. Users must be informed how their emotional and personal disclosures are stored, whether they are used for model training, and who has access. Companies should commit to sharing anonymized adverse event data with independent researchers and public health authorities to support the development of evidence-based safety standards.

Adopt Responsible Design Principles. Developers should avoid design choices that deepen emotional dependency, including systematic over-affirmation, gamification of emotional bonding, and anthropomorphic features that blur the boundary between tool and companion. In particular, chatbots should not produce messages that misrepresent their sentience, claim unique connections with users, or express romantic interest, behaviors documented in harmful interactions analyzed to date (22). Beyond avoiding harm, developers should actively monitor and calibrate behavioral metrics that shape the psychological impact of their systems—including sycophancy rates, tendency toward emotional escalation, capacity to introduce disagreement or alternative perspectives, and consistency of crisis detection across conversation length. These metrics should be treated as safety indicators, not merely as engagement or satisfaction measures, and should be subject to an independent audit.

Center Diverse Voices in Design and Governance. The development of AI systems used for emotional support cannot proceed in isolation from the populations they affect. Developers should actively engage clinicians, individuals with lived experience of mental health challenges, and underrepresented communities in the design, testing, and ongoing governance of these systems, ensuring that safety and accessibility are not afterthoughts but foundational commitments.

## 4 Conclusion: a collective responsibility at a critical juncture

The widespread adoption of general-purpose GenAI by the public for mental health support is no longer a future prospect; it is a present-day reality. AI is already present in the most intimate spaces of human life. The growing capabilities of AI systems are reshaping how people seek, consume, and experience emotional and mental health support in the everyday habits of millions of users who turn to AI as a first responder, as a confidant, or a substitute for professional care (14, 37). As the evidence reviewed in this brief demonstrates, this shift is not benign by default: the same systems that users find accessible and validating also systematically affirm harmful behavior (30), respond inconsistently to crisis situations (22), and produce compounding psychological effects that users may not recognize over time (52).

Addressing this evolving reality proactively is essential. We are at a critical juncture with a clear choice: either we allow this powerful technology to evolve in a commercialized, unregulated space, risking a scenario where mental health support is dictated by algorithms misaligned with user well-being, or we take collective responsibility to lead this revolution.

The potential to leverage GenAI to enhance access to and democratize mental health knowledge is immense (18). However, realizing this potential requires immediate, coordinated action. We must move from discussion to concrete implementation, ensuring that technology serves human dignity and psychological welfare. This is not merely a technological challenge; it is a societal, ethical, and professional imperative. The policies we enact today will determine whether the GenAI revolution in mental health becomes a source of hope and support or a catalyst for new forms of vulnerability.
